# Cell activity during peripheral nerve defect repair process using a nerve scaffold

**DOI:** 10.18632/oncotarget.22978

**Published:** 2017-12-05

**Authors:** Chan Zhou, Jin Li, Huajian You, Jinfeng Lv, Jinlong Yang, Bin Liu

**Affiliations:** ^1^ Chongqing Academy of Animal Science, Chongqing 400015, China; ^2^ Key Laboratory of Freshwater Fish Reproduction and Development, Ministry of Education, School of Life Sciences, Southwest University, Chongqing 400715, China; ^3^ Chongqing Academy of Chinese Materia Medica, Chongqing 400065, China

**Keywords:** fibroin fibre, inflammatory cell, Schwann cell, fibroblast, peripheral nerve defect

## Abstract

Peripheral nerve defects, but not artificial nerves, are repaired by endogenous cells. We examined cell activity during the repair process in the presence of autologous nerves and artificial preparations in order to guide future artificial nerve fabrication. PLGA tubes, nerve scaffolds comprising a PLGA tube plus 6,000 fibroin fibers, or autologous nerves were implanted into 10 mm rat sciatic nerve defects (*n =* 60 per group). Over a period of 1-20 weeks after nerve grafting, sections were stained and imaged to distinguish the cell types present and we quantified the recovery of motor and sensory function in the surgically implanted limb. We observed a decreasing trend in inflammatory cell and fibroblast counts over time which ranked in magnitude as: (PLGA group > nerve scaffold > autologous nerve> sham) and an opposite trend in Schwann cell counts. Differences in withdrawal time from hot water and static sciatic index (SSI) indicated that, after repair, sensory and motor function were best in the sham group, followed by the autologous group, the nerve scaffold group, and the PLGA group. These findings indicate that the inflammatory reaction is significant in the first two weeks after nerve grafting, followed by the rebirth of fibroblasts and Schwann cells, which guide axon regeneration. This inflammatory response was a fundamental stage of peripheral defect repair, but a weaker inflammatory response corresponded to better recovery of sensorimotor functional.

## INTRODUCTION

Peripheral nerve diseases [1–11] commonly seen in the clinic include peripheral nerve sheath tumors [12–15], optic nerve injury [16, 17], peripheral nerve chronic constriction injury [18], and peripheral nerve defects. Clinical [19, 20] and experimental [21–23] research has been carried out to find ways to treat peripheral nerve diseases. Absence of suitable nerve replacement grafts is the main hurdle to improving the low repair rate of peripheral nerve defects. The development of artificial nerve grafts to replace autologous nerve grafts is the focus of considerable current research.

Tissue injury elicits an inflammatory response which has positive attributes for injury repair [24]. Inflammatory responses require inflammatory cells to migrate within and through the vasculature [25]; in addition, integrins are associated with the migration of eosinophils and the activation of macrophages [26], and fibroblasts and Schwann cells are required for nerve regeneration [1, 3, 11]. A peripheral nerve defect actually will be repaired by the patient’s own cells with the assistance of an artificial nerve but not by the artificial nerve itself. Observation of the performance of inflammatory cells, Schwann cells, and fibroblasts in the process of peripheral nerve defect repair will guide the development of future artificial nerve preparations.

To observe the cell performance during the peripheral nerve defect repair with an artificial nerve, a nerve scaffold comprising 6,000 fibroin fibers considered as micro-tracks was prepared, mirroring the 6,000 nerve fibers in rat sciatic nerve. Nerve scaffold, PLGA tube, and autologous nerve were implanted into a 10mm sciatic nerve defect in rats, while a sham operation group with implanted autologous nerve and PLGA tube were used as controls. The recovery of sensory and motor function in surgically implanted limbs were tested according to methods reported in the literature [5, 7]. Inflammatory cells, Schwann cells, and fibroblasts were distinguished by immunofluorescence and hematoxylin and eosin (HE) staining.

## RESULTS

### PLGA and fibroin nerve scaffold can morphologically repair 10 mm rat sciatic nerve defect

**Figure 1:**
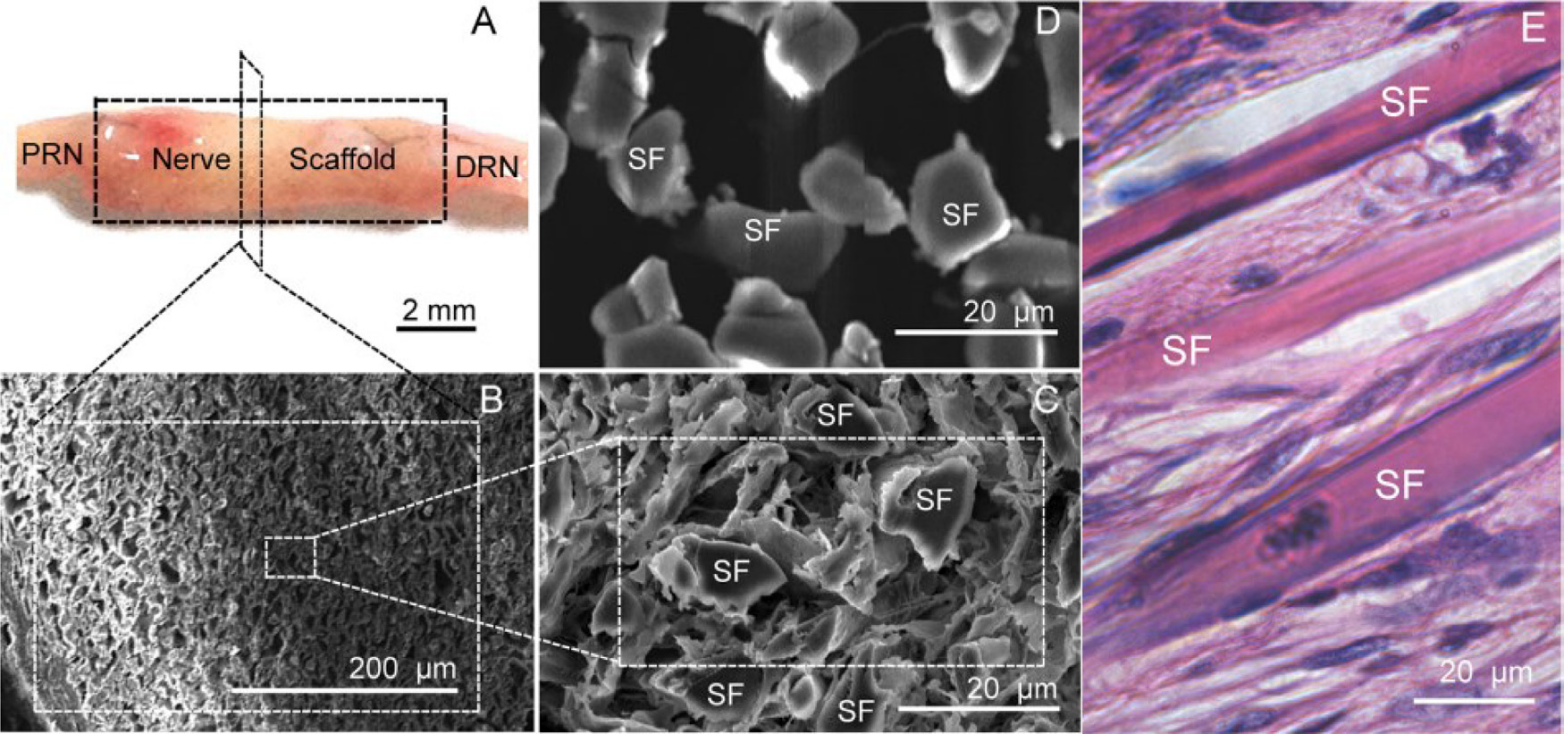
Structure of the nerve scaffold (**A**) Shape of the nerve scaffold after grafting 6 weeks (6 wps). (**B** and **C**) Cross section of the nerve scaffold at 6 (wps) (SEM). (**D**) Cross section of the nerve scaffold before grafting (SEM). (**E**) Longitudinal section of the nerve scaffold at 6 (wps) (HE staining). PRN: The proximal end of the recipient sciatic nerve . DRN: The distal end of the recipient sciatic nerve. SF: Silk fiber.

To observe the result of morphological repair, a nerve scaffold assembled with a PLGA tube and fibroin fibers was grafted to a 10 mm rat sciatic nerve defect. As shown in Figure 1A, at 6 weeks post-grafting (wps), the nerve scaffold achieved proximal and distal fusion with the sciatic nerve in the rat recipients. There were no neoplastic tissues on the graft surface and no adhesions with adjacent muscle tissues, indicating that this nerve scaffold had good biocompatibility.

The sciatic nerve defect ends were bridged and healed, while the adjacent gaps between these fibroin fibers (Figure 1D) were filled by cells (Figure 1B, 1C, and 1E); cells were distributed uniformly along the fibroin fiber (Figure 1E). This indicated that the nerve scaffold fabricated in the present study can morphologically repair the 10 mm rat sciatic nerve defect.

### Inflammatory reaction in grafts were significant in the first two weeks post-surgery

To observe the inflammatory reaction in the nerve scaffold, sections obtained at the first and second week after grafting were stained with immunofluorescence for Integrin-α (red) and a HE staining to display inflammatory cells, while immunofluorescence staining of NGFR P75 (green) and fibronectin (green) was carried out to display Schwann cells and fibroblasts, respectively. The autologous and PLGA tube groups served as controls.

**Figure 2:**
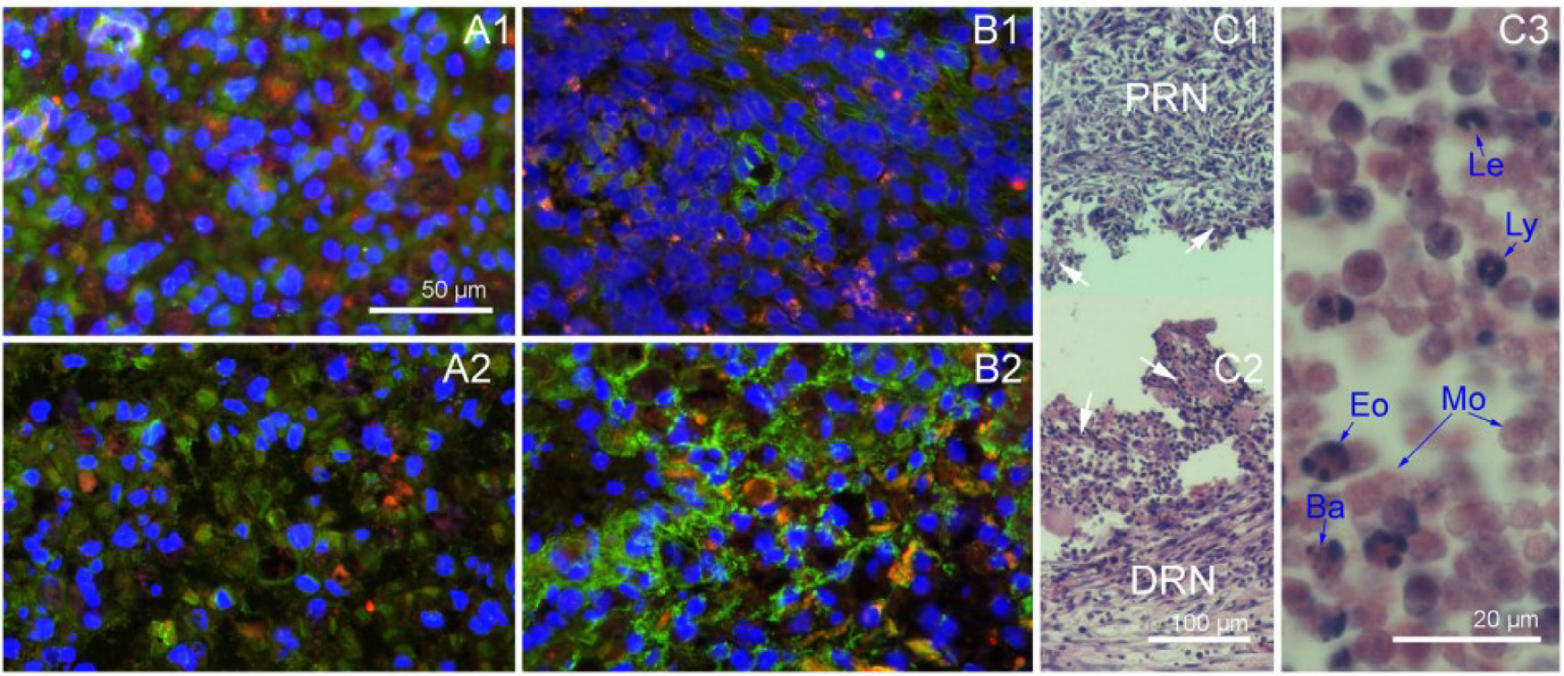
Nerve grafts cross sections obtained in the first two weeks after implantation (**A**1) Autologous sciatic nerve at 1 week, immunofluorescence staining with DAPI (blue), integrin-α(red) and NGFR P75 (green). (**A**2) Autologous sciatic nerve at 2 weeks, immunofluorescence staining with DAPI (blue), integrin-α(red) and NGFR P75 (green). (**B**1) Nerve scaffold at 1 week, immunofluorescence staining with DAPI (blue), integrin-α(red) and fibronectin (green). (**B**2) Nerve scaffold at 2 weeks, immunofluorescence staining with DAPI (blue), integrin-α(red) and fibronectin (green). (**C**1) Longitudinal section of the proximal end of PLGA tube at 2 weeks (HE). (**C**2) Longitudinal section of the distal end of PLGA tube at 2 weeks (HE). (**C**3) A film preparation smeared with the content of PLGA tube at 2 weeks (HE). White arrows: The field of vision show in C3. Ba: Basophile. Eo: Eosinophile. Le: Leukocyte. Ly: Lymphocyte. Mo: Monocyte.

At the first two weeks post-grafting, the cross sections of autologous and nerve scaffold groups were fully filled with cells (Figure 2), while the PLGA tube group was empty (Figure 2C1 and 2C2). The expression of integrin was associated with inflammatory cell activity [11]. Integrin-α immunofluorescence staining results (Figure 2) show that inflammation was significant in the first two weeks post-surgery, but there was a weakened tendency from the first to the second week. Inflammatory cells can be distinguished by the shape of their nuclei. From a film preparation smeared with the content of PLGA tube at 2 weeks post-grafting, the observed inflammatory cells (Figure 2C3) are basophiles, eosinophiles, leukocytes, lymphocytes, and monocytes.

### Observation of Schwann cells, axons, and fibroblasts after grafting

**Figure 3:**
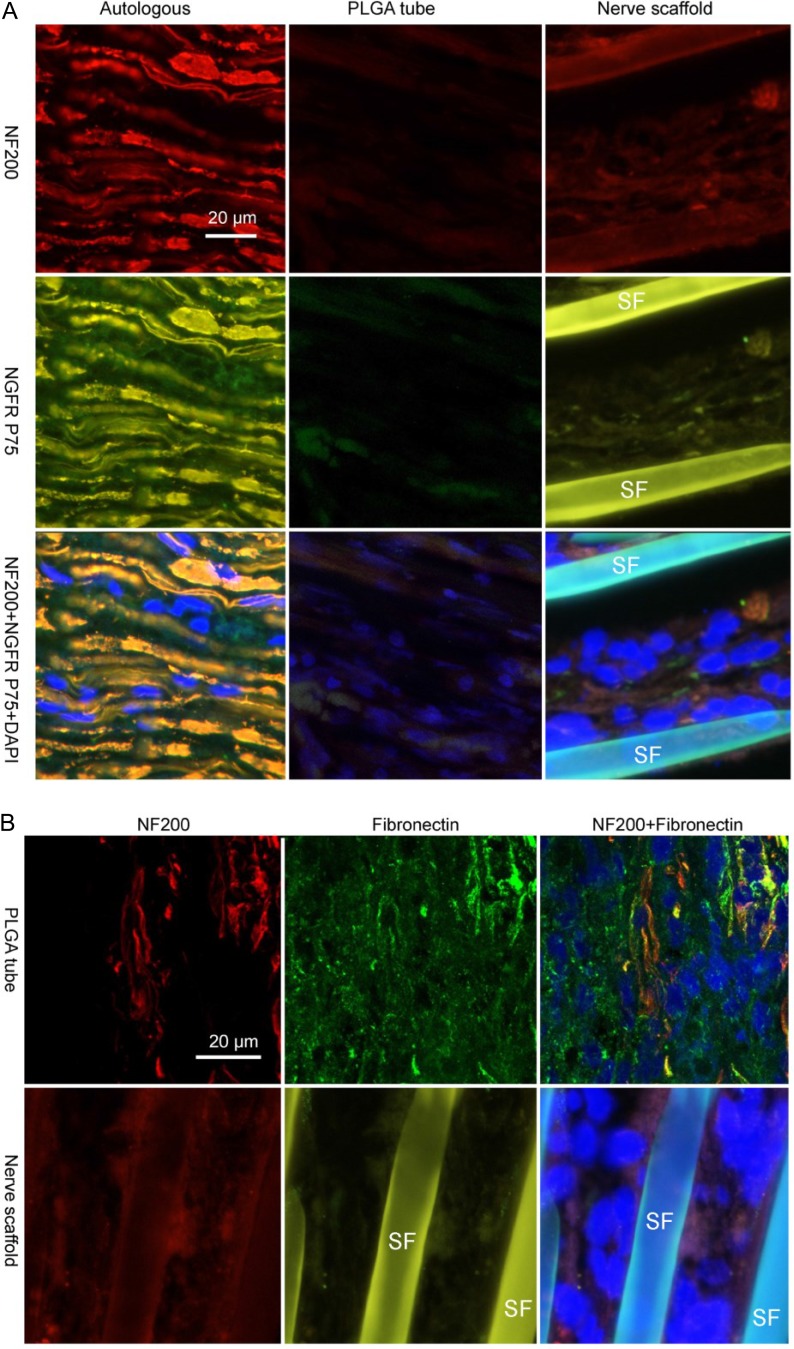

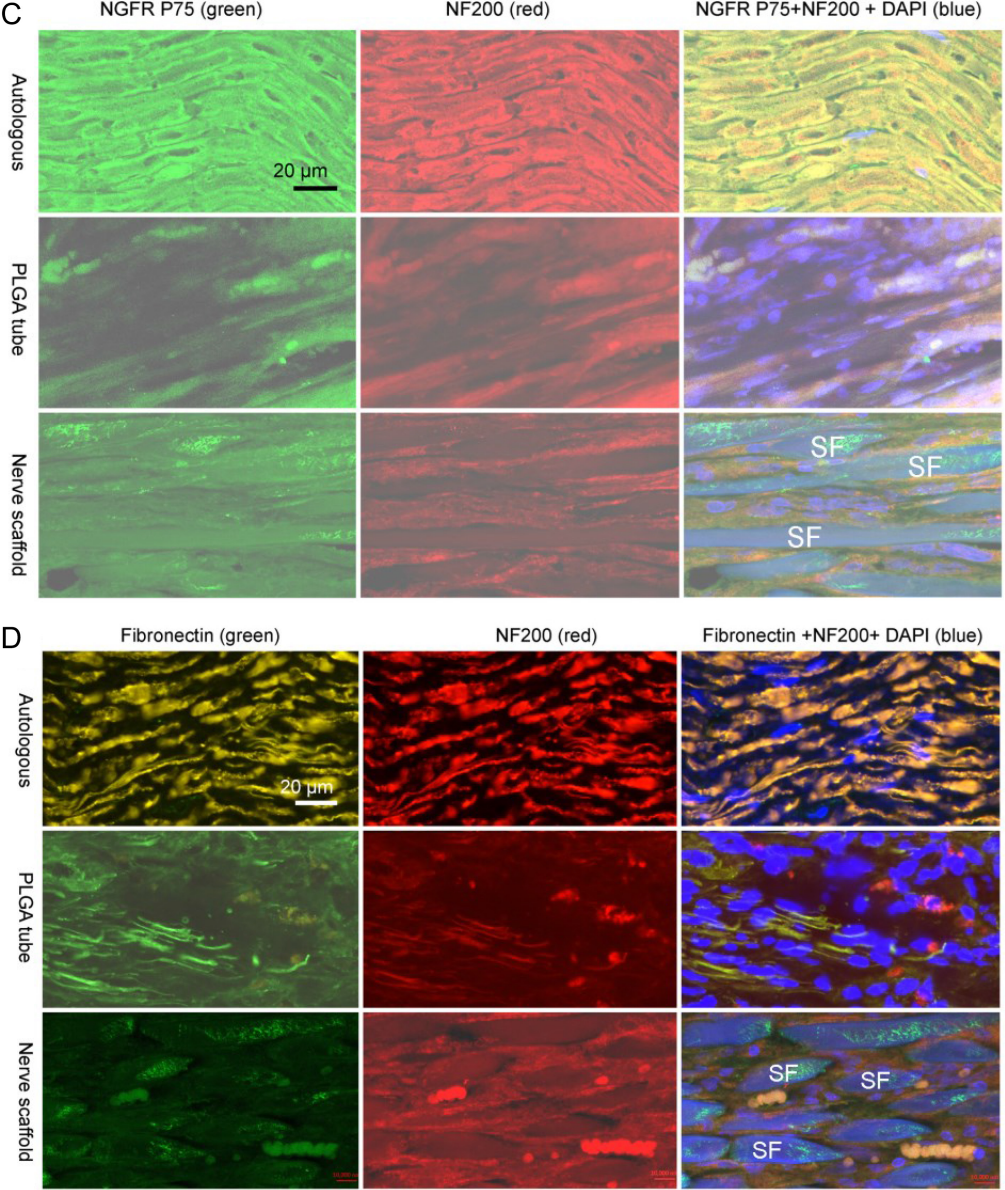
Immunofluorescence staining of nerve grafts frozen longitudinal sections (**A**) Autologous, PLGA tube and nerve scaffold at 3 weeks after implantation were stained with DAPI (blue), NF200 (red), and NGFR P75 (green). (**B**) PLGA tube and nerve scaffold at 3 weeks after implantation were stained with DAPI (blue), NF200 (red), and fibronectin (green). (**C**) Autologous , PLGA tube and nerve scaffold at 20 weeks after implantation were stained with DAPI (blue), NF200 (red), and NGFR P75 (green). (**D**) Autologous , PLGA tube and nerve scaffold at 20 weeks after implantation were stained with DAPI (blue), NF200 (red), and fibronectin (green). SF: Silk fibers.

There were no fibroblasts in the autologous sciatic nerve graft, and fibroblasts were observed at the first week in the nerve scaffold (Figure 2B1–2B2). Schwann cells and axons were observed in sections of PLGA tube and nerve scaffold at the 3rd week after grafting (Figure 3A). There were more axons and Schwann cells in the nerve scaffold than in the PLGA tube. Figure 3B shows that there were more fibroblasts and fewer Schwann cells in the PLGA tube than in the nerve scaffold.

### Fibroblasts can still be observed in the nerve scaffold and PLGA tube 20 weeks after grafting

At 20 weeks post-grafting, a greater number of axons and Schwann cells were observed in the nerve scaffold group (Figure 3C) compared with the PLGA tube group. Fibroblasts were absent on the cross section of the autologous sciatic nerve group. Fewer fibroblasts were observed in the nerve scaffold group (Figure 3D) compared with the PLGA tube group.

### Decreased limb retraction times and increased static sciatic index (SSI) revealed that nerve grafts were repairing peripheral nerve defects

**Figure 4:**
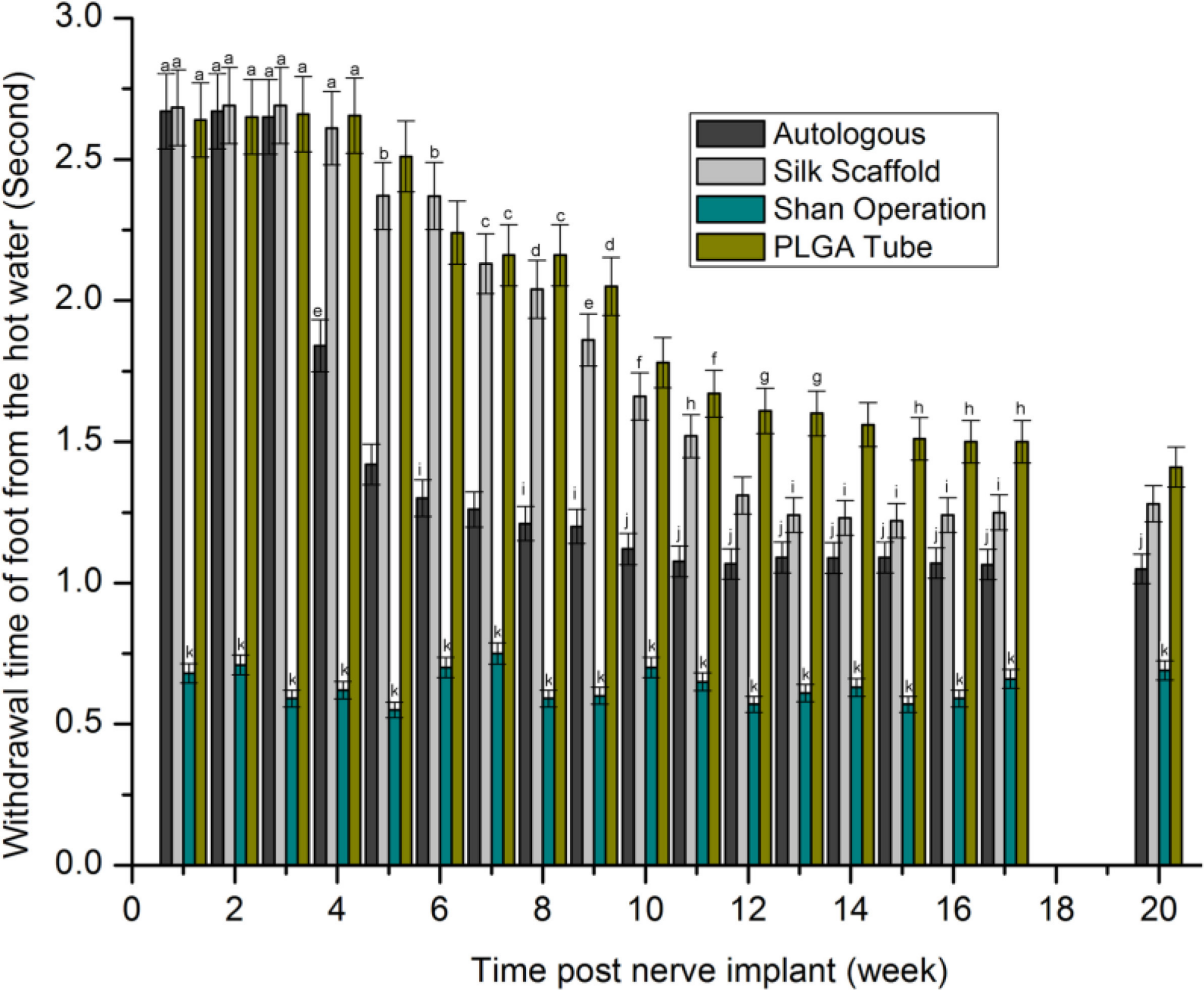
Withdrawal time of foot from hot water (second) after statistical analysis (*p* > 0.05) The values represented with the same characters (a-k) indicated no significant difference, otherwise indicated significant difference.

Our sensory function testing (Figure 4) revealed no significant difference in the time value of the sham operation group with a variance of 0.55–0.75 s, within 4 weeks after nerve grafting. Inter- or intra-group comparison showed a non-significant difference in limb retraction time of approximately 2.65 s. At 5–20 weeks after nerve grafting, with the exception of the sham operation group, values of all groups were reduced over time. There was a significant decreasing trend in limb retraction times from PLGA tube group to nerve scaffold (silk scaffold) group to autologous group to sham operation group.

**Figure 5:**
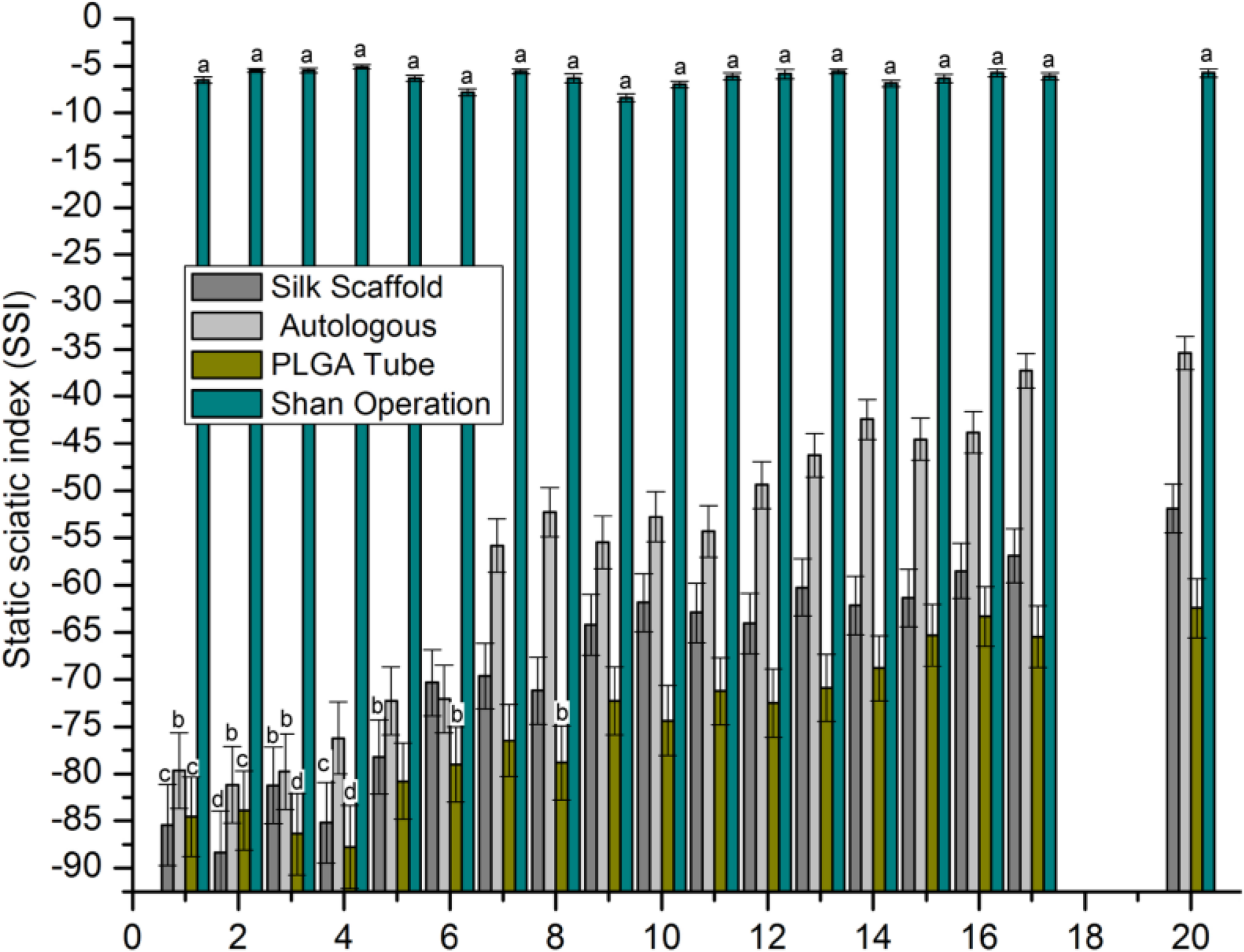
SSI After statistical analysis (*p* > 0.05), the values represented by the same characters (a-d) had no significant difference, all other conditions represent a significant difference.

The motor function testing results (Figure 5) measured as the SSI revealed no significant differences in the sham operation group with a range from -5.1 to -7.8. In the first 4 weeks after nerve grafting, data indicated inconsistency. At 5–20 weeks after nerve grafting, with the exception of sham operation group, values of all groups increased over time. There was a significant increasing trend in SSI from PLGA tube group to nerve scaffold (silk scaffold) group to autologous group to sham operation group.

## DISCUSSION

Observation of cell activity during the process of peripheral nerve defect repair may help guide the preparation of an artificial nerve. A population of 5 types of circulation inflammatory cells was observed in the first two weeks after nerve grafting. This indicated that an inflammatory response was a fundamental stage of peripheral defect repair.

A shorter withdrawal time and a higher SSI value indicated a better repair result for peripheral nerve defect. There was a significant decreasing tendency of surgically implanted limb retraction times which ranked from highest to lowest as PLGA tube group, nerve scaffold group, autologous group and sham operation group, and an increasing trend in SSI which ranked from lowest to highest as PLGA tube group, nerve scaffold group, autologous group, and sham operation group. These results show an increasing degree of peripheral nerve defect repair which ranked (from worst to best) as PLGA tube group, nerve scaffold group, autologous, and sham operation group. There is another decreasing trend in inflammatory response which ranked (from highest to lowest) as PLGA tube group, nerve scaffold group, and autologous group in the first two weeks after nerve grafting. Considering the relationship of inflammatory response and sensorimotor functional recovery, it appears that a weaker inflammatory response corresponded to a better sensorimotor functional recovery. In the first two weeks after nerve grafting the large number of inflammatory cells present and the lack of axonal growth resulted in very little early sensorimotor functional recovery.

Fibroblasts began to appear in the nerve scaffold at the 1st week after grafting and lasted until the 20th week. Fibroblasts appear in the PLGA tube in the 3rd week after grafting. During the observation period of 3–20 weeks, there were more fibroblasts and fewer Schwann cells in the PLGA tube than in the nerve scaffold, corresponding with a worse sensorimotor functional recovery.

Some questions remain to be addressed in the future: 1) to what extent is the inflammatory response beneficial for peripheral nerve repair and how can we regulate it. 2) when and how many fibroblasts appear in an artificial nerve after grafting is proper for peripheral never effect repair and how to control it. 3) How to improve the characteristic of a nerve scaffold comprising micro-channels to achieve a better peripheral nerve effect repair result though it was more effective than a tube.

## MATERIALS AND METHODS

### Preparation of PLGA tube and nerve scaffold

Poly(lactic-co-glycolic acid) or PLGA (80:20, Sigma, USA) was dissolved in chloroform to prepare a 5% (W/V) solution. The resulting solution was placed in a glass petri dish for 12h to form a PLGA film. The resulting film was wound several times around a stick (with an outer diameter of 1.8 mm) to prepare a PLGA tube.

To generate the nerve scaffold, 6,000 fibroin fibers longer than 20 mm were prepared as a bundle, and drawn inside the PLGA tubes with a string. Subsequently, the tubes with the 6,000 fibroin fibers inside were cut to a length of 10 mm, resulting in the generation of a nerve scaffold.

### Animal and nerve graft surgical implantation

Sprague-Dawley rats, weighing 250 ± 10 g, were provided by the Experimental Animal Centre of the Third Military Medical University affiliated to the People’s Liberation Army (PLA), China. One hind limb on each rat was randomly selected for artificial nerve grafting. Preparation of the 10 mm sciatic nerve defect animal model and nerve grafting surgical implantation were carried out in accordance with a previously described method [9]. Briefly, the nerve graft (*n* = 60/group) was implanted to bridge the 10 mm sciatic nerve defect.

### Morphological observation

At specified time points after nerve grafting, the artificial nerve bridging effects were evaluated by observing the graft appearance. Grafts were frozen (-80°C), sectioned (6 µm thickness) using a cryostat (CM1850, LEICA, Germany), stained using immunofluorescent markers or HE, and examined under a laser scanning confocal microscope (LSM200, Zeiss, Germany) to identify different cell types and fibroin fibers within the graft. Nuclei were stained blue using 4’,6-diamidino-2-phenylindole (Biosynthesis Biotechnology, China). Primary antibody for low-affinity nerve growth factor receptor (NGFR) P75 (goat anti-rat; Santa Cruz Biotechnology, USA) was used to identify Schwann cells. Primary antibodies against fibronectin (goat anti-rat; Santa Cruz Biotechnology) and NF200 (rabbit anti-rat; Sigma, USA) were used to identify fibroblasts and axons, respectively. Schwann cells and fibroblasts were labelled green using donkey anti-goat secondary antibody and axons were marked red using donkey anti-rabbit secondary antibody (Life Technologies, USA). Integrin-α(rabbit anti-rat; Santa Cruz Biotechnology, USA ) were used to identify inflammatory cells. Specific steps for immunofluorescence staining were performed according to the manufacturer’s instructions. Some of the grafts were observed with a scanning electron microscope (SEM) (JSM-6510LV, Japan).

### Sensorimotor function testing

In accordance with previous studies [7], all rats were immobilized at 1–20 weeks post-surgery (wps), to ensure the surgically implanted limb was immersed in a hot water bath at a constant temperature of 50°C. The time required to retract the limb in response to this heat stimulus was recorded to assess the recovery of the sensory function in the operated rats. The time of non-surgically implanted limb retraction was also recorded and calculated as an average from the data obtained from all experimental rats: the mean value was considered as the control. In accordance with literature [5], the SSI was measured in rats at 1–20 weeks after surgery to assess the recovery of the motor function in the operated rats.

### Statistical analysis

All motor and sensory function test data were statistically analysed using SPSS software to compare their differences among the different time points in the same group or among groups at the same time point and illustrated using Origin 8.0 software. All graphs were edited for display using Adobe Photoshop 7.0.
